# Supplementary material for: Enhancing Salt Tolerance in Poplar Seedlings through Arbuscular Mycorrhizal Fungi Symbiosis
Source: Plants (Basel). 2024 Jan 14;13(2):233. doi: 10.3390/plants13020233 (PMC10820157; doi:10.3390/plants13020233)
Supplement: Supplementary file 1 [file plants-13-00233-s001.zip › plants-2774587-supplementary.pdf]

Supplementary Materials:

Table S1. Variance analysis of physiological parameters of poplar affected by mycorrhizal inoculation and salt factors.

Index	Significance		
	Mycorrhiza inoculation	Salt stress	Mycorrhiza inoculation×Salt stress
MCR	1.77E-15 ***	0.0041**	0.00012***
SD	<2e-16 ***	0.128	0.323
EEG	5.15E-15 ***	0.0735	0.0708
TG	2.45E-11***	0.922	0.966
P_n	0.0366 *	0.0244 *	0.8834
C_i	0.0179 *	0.0168 *	0.8034
T_r	1.22E-05 ***	0.0943.	0.1749
qN	1.18E-01	0.386	0.87
qP	3.56E-02 *	0.0167 *	0.2975
F_v/F_m	3.38E-04 ***	0.001397 **	0.012069 *
Y(II)	9.44E-04 ***	0.00506 **	0.162554
Shoot biomass	1.50E-06 ***	3.79E-06 ***	5.44E-07 ***
Root biomass	4.32E-09 ***	7.24E-09 ***	1.96E-10 ***
Root/Shoot	3.07E-03**	1.12E-02*	4.46E-05***
RWC	8.20E-02	3.25E-01	4.12E-03**
Lignin	7.84E-09***	1.88E-10***	1.26E-01
Cellulose	3.21E-10***	6.10E-09***	0.606
MDA (Leaf)	9.44E-04***	0.00506***	0.162554***
MDA (Root)	4.70E-05***	4.87E-02*	0.0288*
CAT (Leaf)	2.85E-07***	5.84E-07***	0.266
CAT (Root)	1.08E-02*	1.44E-05***	0.2574
POD (Leaf)	4.95E-13***	< 2e-16***	1.14E-07***
POD (Root)	5.31E-03**	1.85E-10***	6.33E-02.
SOD (Leaf)	8.63E-08***	< 2e-16***	3.72E-06***
SOD (Root)	3.54E-05***	4.40E-13***	9.49E-03***
C (shoot)	1.48E-11***	1.53E-14***	1.27E-05***
C (root)	7.45E-16***	2.43E-13***	8.66E-06***
N (shoot)	2.08E-05***	2.04E-14***	0.33***
N (root)	<2e-16***	<2e-16***	<2e-16***
P (shoot)	1.15E-01	0.0416*	0.7561
P (root)	1.10E-05***	1.34E-07***	0.0538.
Na^+ (shoot)	2.55E-07***	9.56E-08***	8.05E-06***
Na^+ (root)	3.84E-07***	4.14E-11***	1.86E-02*
K^+ (shoot)	2.76E-03**	8.39E-08***	0.7148
K^+ (root)	1.28E-11***	2.83E-13***	2.98E-07***
Ca^{2+} (shoot)	1.10e-07 ***	2.54E-08***	0.00241**
Ca^{2+} (root)	0.00113**	1.47E-13***	6.12e-10***
Mg^{2+} (shoot)	3.98E-01	0.000363***	0.027471*
Mg^{2+} (root)	2.49E-06***	1.17E-11***	8.99E-05***

MCR, Mycorrhizal colonization rate; SD, Spore density; EEG, Easily extractable glomalin-related soil protein; TG, Total glomalin-related soil protein; P_n Photosynthetic rate; C_i, Intercellular CO_2 concentration; T_r, Transpiration rate; qN, Non-photochemical quenching coefficient; qP, Photochemical quenching coefficient; F_v/F_m, Maximum photochemical quantum yield of PSII reaction centers; Y(Ⅱ), Actual quantum yield of PSII; RWC, Relative water content; MDA, Malondialdehyde; CAT, Catalase; POD, Peroxidase; SOD, Superoxide Dismutase; C, Carbon; N,

Nitrogen; P, Phosphorus; Na^+, Sodium ion; K^+, Potassium ion; Ca^{2+}, Calcium ion; Mg^{2+}, Magnesium ion; ns, no significant; *,0.01<p<0.05; **,0.001<p<0.01; ***, p<0.001.

Table S2. Effects of AMF inoculation on plant growth and quality under salt stress.

	P_n (μmol m^{-2} s^{-1})	T_r (mmol m^{-2} s^{-1})	C_i (mmol m^{-2} s^{-1})	qN	qP	F_v/F_m	Y II
N0	35.46±6.98 ab	3±0.21 b	189.47±22.69 b	0.37±0.01 a	0.58±0.06 a	0.77±0.04 b	0.4±0.04 ab
N1	29.41±5.47 b	2.68±0.17 b	148.83±30.89 bc	0.29±0.05 a	0.56±0.07 ab	0.74±0.06 b	0.39±0.02 b
N2	26.62±3.91 b	2.19±0.13 b	162.08±32.03 c	0.23±0.06 a	0.55±0.06 b	0.62±0.04 c	0.32±0.03 c
A0	38.88±1.33 a	3.69±0.38 a	230.85±6.21 a	0.37±0.06 a	0.64±0.07 a	0.83±0.01 a	0.49±0.05 a
A1	34.21±3.14 ab	3.78±0.37 b	171±34.38 a	0.35±0.06 a	0.61±0.03 a	0.76±0.03 b	0.42±0.02 ab
A2	32.48±1.35 b	3.63±0.5 b	197.77±14.7 a	0.32±0.03 a	0.57±0.04 ab	0.78±0 b	0.42±0.03 ab

RWC, Relative water content; WSD, Water saturation deficit; N0, N1, N2: No stress, 100 mM salt stress and 200 mM salt stress without AMF inoculation. A0, A1, A2: No stress, 100 mM salt stress and 200 mM salt stress under AMF inoculation. Different letters on top of the bars indicate significant differences ($p < 0.05$). All data are presented as means ± standard errors.

Table S3. Effects of AMF inoculation on photosynthesis and chlorophyll parameters of poplar under different salt stress.

	Shoot weights (g)	Root weights (g)	Root/Shoot	RWC	WSD	Lignin (mg·mL^{-1})	Cellulose (mg·mL^{-1})
N0	5.89±1 b	14.3±0.14 c	0.41±0.02 b	0.73±0.07 a	0.27±0.07 bc	12.4±0.35 d	10.12±0.33 c
N1	4.06±1.4 c	10.0±0.54 d	0.4±0.02 b	0.68±0.02 b	0.32±0.02 b	14.02±0.22 c	12.74±0.72 b
N2	2.22±0.92 d	6.54±0.22 e	0.34±0.03 c	0.63±0.02 c	0.32±0.02 a	15.58±0.34b	15.76±0.64 a
A0	8.47±0.29 a	17.6±0.19 a	0.48±0.01 a	0.79±0.05 a	0.21±0.05 c	14.16±0.06 c	15.18±0.72 e
A1	6.81±0.98 b	14.7±0.33 b	0.46±0.02 a	0.74±0.05 b	0.26±0.05 bc	15.59±0.13 b	18.23±0.06 d
A2	3.23±0.95 c	8.7±0.13 d	0.37±0.03 bc	0.71±0 c	0.29±0 bc	16.77±0.05 a	20.57±0.69 c

P_n Photosynthetic rate; T_r, Transpiration rate; C_i, Intercellular CO_2 concentration; qN, Nonphotochemical quenching coefficient; qP, Photochemical quenching coefficient; F_v/F_m, Maximum photochemical quantum yield of PSII reaction centers; Y(II), Actual quantum yield of PSII; Different letters on top of the bars indicate significant differences ($p < 0.05$). All data are presented as means ± standard errors.
